# Evaluating Manganese, Zinc, and Copper Metal Toxicity on SH-SY5Y Cells in Establishing an Idiopathic Parkinson’s Disease Model

**DOI:** 10.3390/ijms242216129

**Published:** 2023-11-09

**Authors:** Sahar H. Pradhan, James Y. Liu, Christie M. Sayes

**Affiliations:** Department of Environmental Science, Baylor University, Waco, TX 76798, USA; sahar_pradhan@baylor.edu (S.H.P.);

**Keywords:** SH-SY5Y, hydroxydopamine, dose–response curves, neurodegeneration, occupational exposure

## Abstract

Parkinson’s disease (PD) is a neurodegenerative condition marked by loss of motor coordination and cognitive impairment. According to global estimates, the worldwide prevalence of PD will likely exceed 12 million cases by 2040. PD is primarily associated with genetic factors, while clinically, cases are attributed to idiopathic factors such as environmental or occupational exposure. The heavy metals linked to PD and other neurodegenerative disorders include copper, manganese, and zinc. Chronic exposure to metals induces elevated oxidative stress and disrupts homeostasis, resulting in neuronal death. These metals are suggested to induce idiopathic PD in the literature. This study measures the effects of lethal concentration at 10% cell death (LC_10_) and lethal concentration at 50% cell death (LC_50_) concentrations of copper, manganese, and zinc chlorides on SH-SY5Y cells via markers for dopamine, reactive oxygen species (ROS) generation, DNA damage, and mitochondrial dysfunction after a 24 h exposure. These measurements were compared to a known neurotoxin to induce PD, 100 µM 6-hydroxydopamine (6-ODHA). Between the three metal chlorides, zinc was statistically different in all parameters from all other treatments and induced significant dopaminergic loss, DNA damage, and mitochondrial dysfunction. The LC_50_ of manganese and copper had the most similar response to 6-ODHA in all parameters, while LC_10_ of manganese and copper responded most like untreated cells. This study suggests that these metal chlorides respond differently from 6-ODHA and each other, suggesting that idiopathic PD utilizes a different mechanism from the classic PD model.

## 1. Introduction

Parkinson’s disease (PD) is a progressive neurodegenerative disorder characterized by a decline in movement coordination and cognitive impairment. According to global estimates from the World Health Organization, over 8.5 million individuals were diagnosed with PD in 2019 [1]. The worldwide prevalence of PD is likely to exceed 12 million cases by 2040 [2]. While hereditary factors contribute to 3–5% of cases, most cases are linked to idiopathic (or sporadic) origins [3]. The onset of idiopathic PD is attributed to three key factors: age, genetics, and environmental influences [4]. A primary focus of environmental influences includes metal-induced PD from occupational exposure. While there are clinical cases of increased occupational risk with idiopathic PD, there is no direct link to connect the two. Instead, a substantial body of literature highlights the impact of commonly utilized metals in the agriculture, manufacturing, and mining industries and higher clinical cases of PD or PD-associated symptoms [5,6,7]. The underlying mechanism of metal-induced degeneration is unclear; some highlighted mechanisms are oxidative stress, dopamine deficiency, and reduced adenosine triphosphate (ATP) production [7,8]. Dopamine degeneration occurs in the substantia nigra, leading to deficiency and eventual hallmark symptoms such as motor impairments and cognitive decline. Mechanisms inducing dopaminergic cell death have been suggested to be motivated by oxidative stress, excitotoxicity, mitochondrial dysfunction, DNA fragmentation, and protein misfolding [9,10]. 

Research focused on understanding metal-induced neurotoxicity and its long-term impacts is still ongoing. Prolonged exposure to heavy metals is reported to induce or progress muscular and neurological degenerative conditions such as PD, muscular dystrophy, and Alzheimer’s disease [11]. The metals linked to PD pathophysiology include lead (Pb), mercury (Hg), copper (Cu), manganese (Mn), aluminum (Al), iron (Fe), and zinc (Zn) [12]. While the brain’s selective barrier can protect against the uptake of lipophilic and large particles, heavy metals exploit alternative mechanisms, allowing them to cross the membrane [13,14]. The largest concentration of copper, zinc, and manganese is in the brain, where these metals significantly contribute to neuronal functions [15]. These biological processes include facilitating electron transfer, supporting synaptic transmission, energy generation, and preventing oxidative stress [15,16]. Therefore, external exposure to these metals can easily bypass protective barriers such as the blood–brain interface. 

Abnormal copper, zinc, and manganese accumulation and dispersion have been observed in PD patients. Analysis of post-mortem brain tissue from 13 PD patients indicated the compartmentalization of these metals in the substantia nigra (SN), the primary site of dopaminergic degeneration [15,17]. This highlights the increased risk of occupational exposure in maintaining strict regulation of metal toxicity. Additionally, elevated oxidative stress, increased superoxide dismutase (SOD) activity, and disrupted metal homeostasis have been observed in the blood and serum of welders exposed to these three metals [18]. Copper, zinc, and manganese have been associated with similar biomarkers associated with PD pathology, including generating reactive oxygen species (ROS), disruptions in mitochondrial function, DNA damage, and cellular apoptosis [19]. Specifically, manganese and zinc have been considered models for PD in mice inhalation studies due to their ability to mimic the disease conditions [20,21].

Current methods of assessing neurotoxicity rely on ethically debated, time-consuming, and expensive in vivo models; therefore, study designs employing high-throughput, characterized in vitro models can provide significant preliminary insights. In vitro models have helped to elucidate the cellular mechanisms for PD development, often utilizing neurotoxins to mimic disease characteristics. Commonly used neurotoxins include 6-hydroxydopamine (6-ODHA) and 1-methyl-4-phenyl-1,2,3,5-tetrahydropyridine (MPTP) [22,23]. 6-ODHA targets dopaminergic neurons via oxidation to induce cytotoxicity [24]. Additionally, 6-ODHA is linked to mitochondrial disruption, which allows our study to assess comparative mitochondrial responses to metal-induced neurotoxicity [23,25]. 

This study utilizes the SH-SY5Y cell line to assess neurotoxicity; these cells are a sub-cloned line derived from a neuroblastoma cell and serve as a model for neurodegenerative disorders [26]. These cells are widely utilized as dopaminergic cell models for PD and express *PARK2*, *PINK1*, *LRRK*, and *SNCA*, PD-associated genes [27,28,29]. An acute treatment (24 h) of 100 µM 6-ODHA was used to replicate PD conditions and measured against sublethal (10% cytotoxicity; LC_10_) and lethal (50% cytotoxicity; LC_50_) concentrations of manganese (II) chloride (MnCl_2_), zinc (II) chloride (ZnCl_2_), and copper (II) chloride (CuCl_2_). LC_10_ mimics a single acute exposure, while LC_50_ corresponds to “lifetime” exposure. Oxidative stress, dopamine concentration, and DNA damage were assessed as primary endpoints for metal-induced toxicity and PD. Additionally, mitochondrial function was evaluated as a secondary endpoint. This study seeks to elucidate whether idiopathic metal-induced neurodegeneration operates through similar pathways as a well-established chemically induced PD model or whether these metals employ alternative mechanisms to trigger neurotoxicity.

## 2. Results

**Figure 1:**
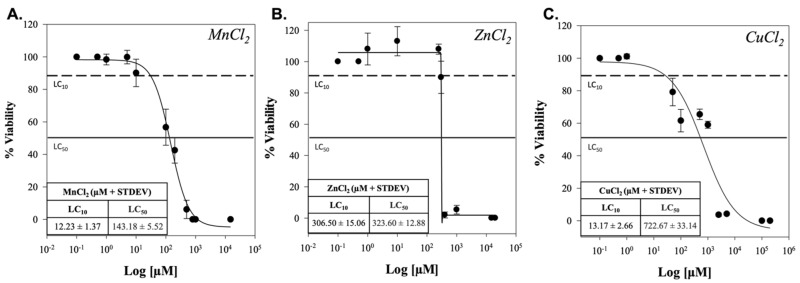
Dose–response curves for three chloride metals, (**A**) manganese chloride, (**B**) zinc chloride, and (**C**) copper chloride on SH-SY5Y cells for a 24 h exposure. Viability was determined via normalization against untreated cells. LC_10_ was determined to be a “sublethal concentration” (dotted), and LC_50_ was determined to be a “lethal concentration” (solid). The molarity equivalent of concentration is displayed in the left corner of each respective graph.

Dose–response (D/R) curves were utilized to extrapolate sublethal (lethal concentration of 10%; LC_10_) and lethal (lethal concentration of 50%; LC_50_) concentrations of manganese chloride, zinc chloride, and copper chloride after 24 h of exposure to the SH-SY5Y cells (Figure 1). Manganese chloride was the most cytotoxic, with an LC_10_ at 12.23 ± 1.37 µM and LC_50_ at 143.18 ± 5.52 µM. Zinc chloride resulted in the steepest transition between sublethal and lethal responses, with an LC_10_ at 306.50 ± 15.06 µM and LC_50_ at 323.60 ± 12.88 µM. The least cytotoxic response was from copper chloride with an LC_10_ at 13.17 ± 2.66 µM and LC_50_ at 722.67 ± 33.14 µM. D/R measurements are reported in Supplemental Table S1.

**Figure 2:**
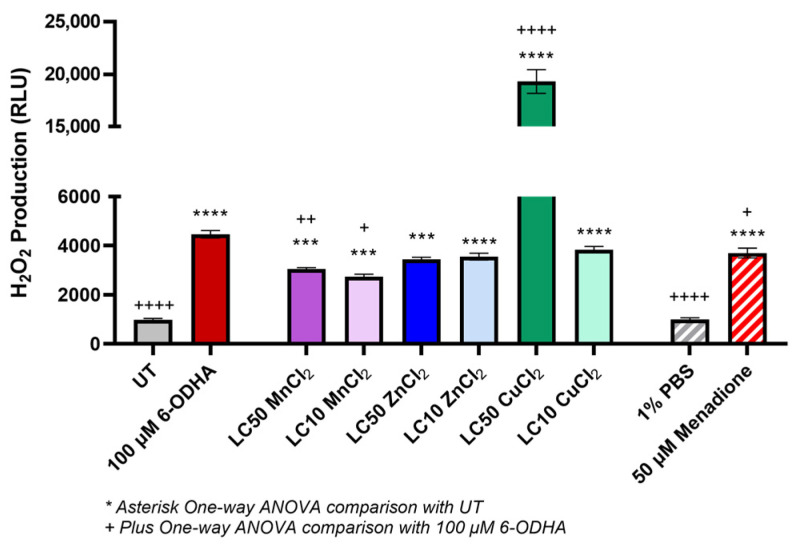
Relative H_2_O_2_ production was measured after a 24 h exposure to MnCl_2_, ZnCl_2,_ and CuCl_2_ and 100 µM 6-ODHA. As a positive control, 50 µM menadione was used, and 1% PBS was used as a negative control. After 24 h, copper at lethal and sublethal concentrations had the highest effect, then zinc, and lastly, manganese at only the lethal concentration resulted in elevated ROS compared to the untreated (UT). Statistical significance codes: treatment vs. untreated control (UT, *) and positive control (6-ODHA, +). Asterisks or plus signs indicate *p*-value ranges: ^+^ 0.01–0.05, ^++^ 0.001–0.01, *** 0.0001 to 0.001, ****^,++++^ <0.0001.

The production of H_2_O_2_ from SH-SY5Y cells after 24 h exposure to MnCl_2_, ZnCl_2_, and CuCl_2_ was assessed against the 6-ODHA model for Parkinson’s and untreated cells (Figure 2). Compared to untreated cells (UT), 100 µM 6-ODHA produced a significant amount of H_2_O_2_ (4466.75 ± 149.77 relative luminescence units (RLU)). Among the metal chlorides, zinc at sublethal and lethal concentrations resulted in H_2_O_2_ generation not significantly different from 6-ODHA (3440.25 ± 933.91 and 3556.5 ± 140.8036 RLU, respectively). The LC_50_ of copper resulted in exceedingly high and significant production of ROS compared to the UT and 6-ODHA groups (19,310 ± 1127.32 RLU). In the absence of cells, copper also resulted in a high production of H_2_O_2_, suggesting that the metal chloride itself is inducing ROS and not the cellular interaction alone. The LC_10_ of copper interaction with SH-SY5Y produced H_2_O_2_ levels similar to 6-ODHA after a 24 h exposure (3840 ± 124.88 RLU). Manganese at sublethal and lethal concentrations generated significant H_2_O_2_ compared to UT (2739.75 ± 98.2 and 3055.5 ± 54.03 RLU, respectively) but not the same magnitude as the other metal chlorides compared to 6-ODHA. 

**Figure 3:**
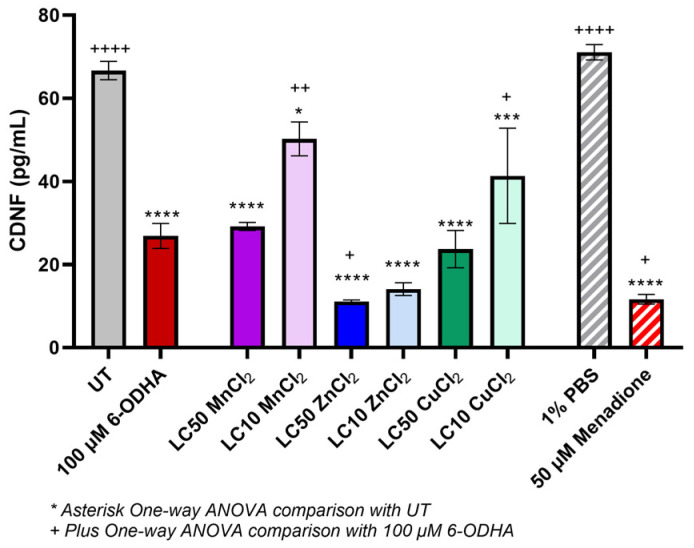
Conserved dopamine neurotrophic factor (CDNF) concentration (pg/mL) after 24 h exposure to MnCl_2_, ZnCl_2_, and CuCl_2_ and 100 µM 6-ODHA. As a positive control, 50 µM menadione was used, and 1% PBS was used as a negative control. After 24 h exposure, ZnCl_2_ at lethal and sublethal concentrations had the highest effect, then CuCl_2_ and MnCl_2_. Statistical significance codes: treatment vs. untreated control (UT, *) and positive control (6-ODHA, +). Asterisks or plus signs indicate *p*-value ranges: *^,+^ 0.01–0.05, ^++^ 0.001–0.01, *** 0.0001 to 0.001, ****^,++++^ <0.0001.

The protection of dopaminergic cells after exposure to the metal chlorides and 6-ODHA was assessed via conserved dopamine neurotropic factor (CDNF) concentration, where a reduction in CDNF concentration corresponds to a decrease in dopamine (Figure 3). After exposure to 100 µM 6-ODHA (26.90 + 2.99 pg/mL), dopamine was significantly reduced compared to the UT (66.68 + 2.21 pg/mL). Among the metal chlorides, zinc at lethal and sublethal concentrations had the most significant loss of dopamine even compared to 6-ODHA (11.0885 + 0.39 pg/mL and 14.08 + 1.53 pg/mL, respectively). The LC_50_ of copper and manganese had similar losses of dopamine compared to 6-ODHA. In contrast, the LC_10_ of manganese and copper did not result in the same response of CDNF loss (approx. ~30–40% reduction), which was not statistically different from 6-ODHA. All metal chlorides significantly lost dopamine compared to the untreated cells (indicated as UT). 

**Figure 4:**
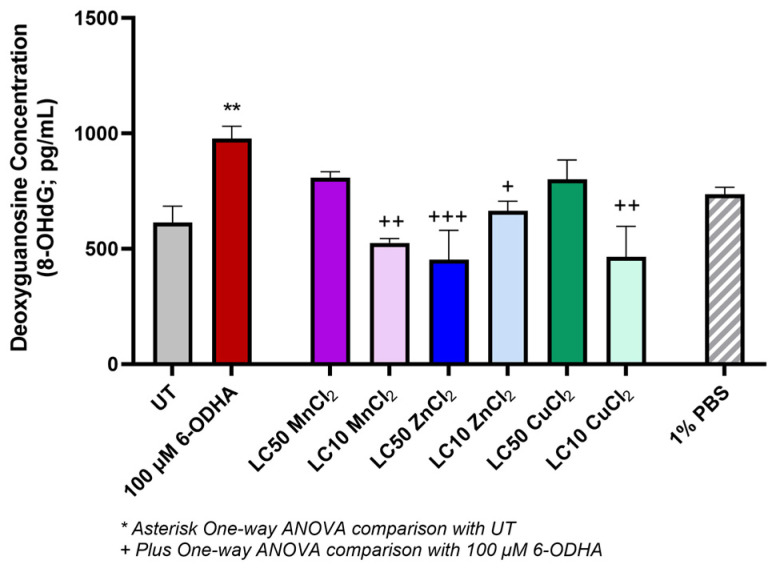
8-hydroxy-2’-deoxyguanosine (8-OHdG) species were measured in digested DNA samples (pg/mL) after exposure to MnCl_2_, ZnCl_2_, and CuCl_2_ and 100 µM 6-ODHA. Use of 1% PBS as a negative control. After 24 h exposure, LC_50_ concentrations of MnCl_2_ and CuCl_2_ induced higher concentrations of 8-OHdG indicative of DNA damage. Statistical significance codes: treatment vs. untreated control (UT, *) and positive control (6-ODHA, +). Asterisks or plus signs indicate *p*-value ranges: ^+^ 0.01–0.05, **^,++^ 0.001–0.01, ^+++^ 0.0001 to 0.001.

The concentration of 8-hydroxy-2’-deoxyguanosine (8-OHdG) species was measured as a response to identifying DNA damage on digested DNA samples (Figure 4). 8-OHdG is also a ubiquitous marker for oxidative stress. After exposure to 100 µM 6-ODHA (978.02 ± 53.5 pg/mL), there was a significantly increase in 8-OHdG compared to the UT (614.41 ± 70.39 pg/mL). Lethal concentrations of MnCl_2_ (807.76 ± 26.35 pg/mL) and CuCl_2_ (800.34 ± 84.83 pg/mL) induced responses similar to 6-ODHA, though this increase was not statistically significant. Sublethal concentrations of MnCl_2_ (525.87 ± 18.58 pg/mL) and CuCl_2_ (466.23 ± 131.71 pg/mL) did not induce any significant 8-OHdG production compared to the UT. Zinc (sublethal and lethal concentrations) indicated no DNA damage (>670 pg/mL).

**Figure 5:**
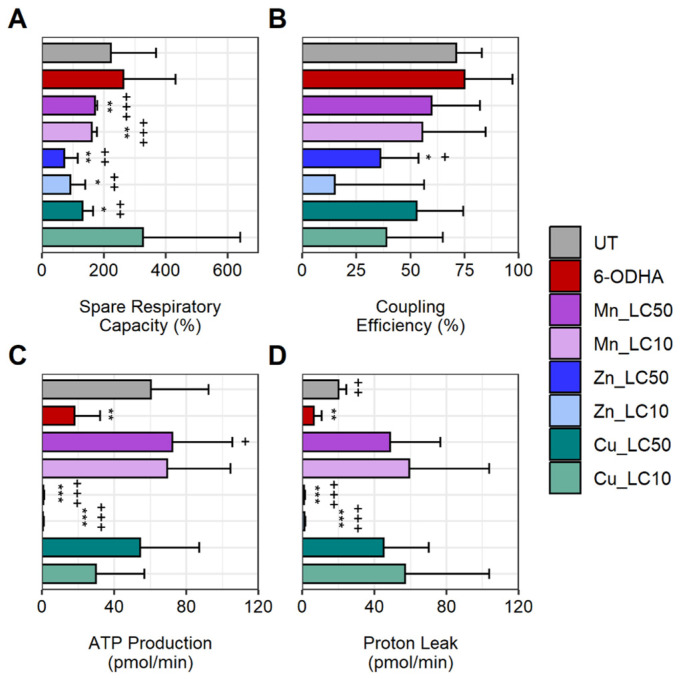
Mitochondrial respiration functional parameters were assessed using alterations in the ETC. (**A**) Spare respiratory capacity (%), which measures the difference between maximal respiration and basal respiration; (**B**) coupling efficiency (%) by measuring ATP production per available oxygen; (**C**) ATP production; and (**D**) proton leak across ETC. Statistical significance codes: treatment vs. untreated control (UT, *) and positive control (6-ODHA, +). Asterisks or plus signs indicate *p*-value ranges: *^,+^ 0.01–0.05, **^,++^ 0.001–0.01, ***^,+++^ 0.0001 to 0.001.

Mitochondrial dysfunction was assessed using four functional parameters extrapolated from real-time oxygen consumption data (Supplemental Figure S1). The parameters comprised spare respiratory capacity (SR), coupling efficiency (CE), ATP production, and proton leak (Figure 5). SR measures the difference between the maximum and basal oxygen consumption rates. While 100 µM 6-ODHA did not alter the SR, all metal chlorides (except LC_10_ CuCl_2_) significantly reduced the SR by ~1/3 of total capacity compared to the UT. CE measured ATP production per available oxygen and was only altered by a lethal concentration of ZnCl_2_. ATP production was significantly reduced by exposure to 100 µM 6-ODHA (18 ± 14.24 pmol/min; UT 60.4 ± 31.9 pmol/min). MnCl_2_ and CuCl_2_ did not affect ATP production. Sublethal and lethal concentrations of ZnCl_2_ significantly decreased ATP production (<1 pmol/min) compared to the UT and 100 µM 6-ODHA. Proton leak measures uncoupled protons and can indicate mitochondrial damage. MnCl_2_ and CuCl_2_ induced high proton leaks when compared to UT. After exposure to ZnCl_2_, the SH-SY5Y completely shut down the electron transport chain (ETC) (Supplemental Figure S1), producing no ATP nor proton leak. The ratio of proton leak to ATP production for 6-ODHA was ~0.45 pmol/min compared to the UT, ~0.17 pmol/min.

**Figure 6:**
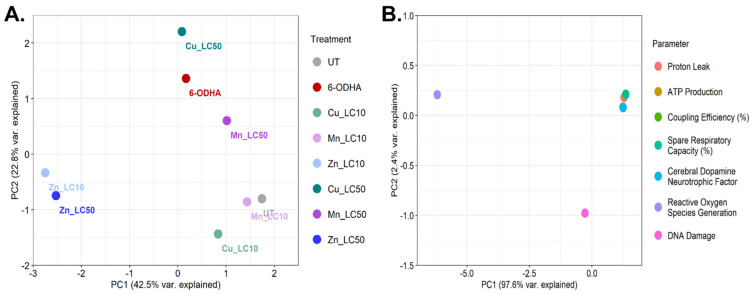
Principal component analyses by (**A**) treatment and (**B**) toxicological parameters, performed using R. Clustered treatments are more similar to variance in parameters and vice versa. The percent variance explained is provided for the two largest principal components. Untreated cells are labeled as UT.

Principal component analysis (PCA) was conducted across all treatments and seven parameters (Figure 6). The parameters chosen were the four related to mitochondrial respiration and one each for CDNF concentration, ROS, and DNA damage. Among the treatments, the LC_10_ and LC_50_ for zinc were closely clustered, as were the UT and LC_10_ for manganese. Notably, only 65% of the variance was explained by principal component 1 (PC1) and principal component 2 (PC2) so that additional separation may occur in higher dimensions. For the parameters, nearly all the variance was concentrated in PC1; most parameters clustered closely, while ROS and DNA damage were distinct.

## 3. Discussion

Heavy metal exposure in environmental and occupational settings continues to pose a significant global health threat. Manganese, zinc, and copper are all considered trace metals and are naturally found in the environment at low levels, but with heavy exposure, these metals induce toxic effects. Chronic exposure to manganese, zinc, and copper increase risk factors for idiopathic Parkinson’s or the development of PD-like symptoms (e.g., cognitive decline, behavioral alterations, and motor deficits). These metals pose an occupational threat to welders, as these metals are generated in welding fumes and miners [18,30,31,32]. In particular, a high risk for manganese-induced parkinsonism has been linked to agriculture workers utilizing manganese-based pesticides and fungicides [30,31,33]. Applying these pesticides/fungicides using aerial and ground treatments threatens inhalation and ingestion via contaminated drinking wells [33,34]. Non-lethal and lethal concentrations were used to represent single and lifetime exposures accurately.

Dose–response testing indicated manganese had the most cytotoxic response, while copper was the least cytotoxic. Interestingly, zinc had a very steep response pattern, where the difference between the LC_10_ and LC_50_ was very close in concentration (µM). This pattern is seen with zinc oxide (ZnO) particles in A549, BEAS-2B, and Ana-1 cells [35,36]. Metal accumulation tends to target three different aspects leading to pathology: 1. increased oxidation, 2. reduced ATP synthesis, and 3. increased dopamine metabolism [37,38]. These mechanisms were measured via H_2_O_2_ production, DNA damage, oxygen consumption efficiency, and CDNF concentration.

The specific loss of dopaminergic cells is a crucial element for PD pathology. CDNF concentration is used to determine the protection of dopaminergic neurons against toxic damage [39]. After 24 h of metal exposure, manganese (LC_50_) and copper responded similarly to 6-ODHA, while zinc significantly depleted dopamine. Lifetime occupational exposure to copper and manganese can manifest Parkinson-like symptoms and increase the overall risk of PD 2-fold to 10-fold [37]. While the mechanisms of zinc-associated PD are poorly understood, it is known that Zn^2+^ is taken up into neurons via the glutamate membrane receptors and that Zn^2+^ acts synergically with dopamine by inducing high levels of oxidative stress, which was noted in ROS generation [40,41,42]. This mechanism may induce dopaminergic cell death and lead to the depletion of dopamine measured.

Associated pathways inducing dopaminergic cell death include oxidative stress and DNA damage, measured via H_2_O_2_ production and 8-OHdG, respectively [43,44,45,46,47]. A major source of ROS is transition metals, which have been linked to PD pathology via ROS-mediated mechanisms, which was seen with manganese exposure [48]. Copper-induced substantial H_2_O_2_ production which is seen in cell-free conditions, with preliminary studies and literature assessing copper exposure and amyloid deposits for Alzheimer’s [49]. None of the treatments, except 6-ODHA, induced DNA damage, measured via 8-OHdG. This biomarker evaluates oxidative DNA damage and PD progression [50,51,52]. This response suggests that metal-generated oxidative damage is not significant enough to lead to permanent DNA damage at this exposure timepoint. This is possible if the exposure timepoint does not extend out enough to lead to this damage, such as a 48 to 72 h exposure for these cells, as measured using comet assay with Mn^2+^ in another study, other factors influencing the data measured are possible antioxidant or neuroprotective measures suggesting no DNA damage has occurred [53,54].

Neuronal function heavily depends on mitochondrial activity and integrity due to its high energetic demands. Mitochondrial dysfunction can attenuate cellular stress and promote dopaminergic cell death [55]. One of the critical endpoints to identify whether neurons are maintaining their energetic demands is to measure ATP production [56]. This study assessed mitochondrial health utilizing four parameters: ATP production, spare respiratory capacity (SR), coupling efficiency, and proton leak. According to the live metabolic data, one of the significant findings was the profound effect zinc had on the ETC at LC_10_ and LC_50_. This finding supports the synergistic effect between Zn^2+^ and dopamine-inducing cellular disruption. This response has also been noted in ETC studies on rat brains showing inhibited oxygen consumption after zinc exposure [42,57]. The parameter SR defines the mitochondrial “energy reserves” [58]. A reduced SR suggests an inability for cells to meet the energetic demands during cellular stress, which was seen with all metal chloride exposure except for the LC_10_ of copper [59]. These data corroborate mitochondrial respiration studies, which report heavy metal exposure inducing repression of energy reserves in SH-SY5Y cells [60,61]. The next parameter measured was proton leak, which quantifies non-ATP-producing protons. The results indicated that manganese and copper increased proton leak above untreated cells, consistent with the current literature [62]. Zinc indicated a reduction in proton leak, which could result from the detrimental effects induced by zinc on the ETC at a 24 h exposure. The coupling efficiency did not indicate any significant changes.

PCA was utilized in the study to compare the weight of evidence among the seven datasets and eight different exposures. While zinc showed the most potent effect out of the three divalent metals, the PCA indicates that it may have dissimilar modes of action compared to 6-ODHA. While not strikingly similar, the high doses of copper and manganese may align better with the effects of 6-ODHA. Regarding the different methods employed in this study, CDNF concentration appears to have strong collinearity with mitochondrial respiration endpoints. They might be connected mechanistically within the scope of divalent metals and the cell model used. Meanwhile, ROS and DNA damage may be needed to differentiate responses that appear similar using the other endpoints. Some limitations of the study that might alter the weight of evidence would be that our model utilizes one cell line at one exposure timepoint, limiting our ability to account for biological response mechanisms, such as the complexity seen with our oxidative stress measurements. Measurements at 24 h affect the ability to properly compare LC_10_ and LC_50_ responses, as sublethal measurements extend to 72 h. This study’s post-exposure period was 24 h to reduce confounding factors such as potential recovery. Other limitations include that the LC_50_ and LC_10_ for zinc showed an extremely steep response pattern and higher potential for error. Future studies should include microglia for in vitro analyses, as the current literature suggests their protective role in mediating cytotoxicity, neurodegeneration, and increased signals at extended exposure times.

## 4. Materials and Methods

### 4.1. Chemicals and Reagents

All metal salts were purchased at the highest quality (≤97%) for analysis: manganese (II) chloride (MnCl_2_; #244589, Sigma-Aldrich, Saint Louis, MO, USA), copper (II) chloride (CuCl_2_, #270532500, Acros Organics, Geel, Belgium), and zinc (II) chloride (ZnCl_2_, #A16281, Thermo Fisher Scientific, Waltham, MA, USA). 6-hydroxydopamine hydrochloride (6-ODHA; H4381, Sigma Aldrich) was a positive control for a neurodegenerative disease state. All salts, including the positive control, were solubilized in 1 mL of ultrapure water and then diluted to working concentrations in cell culture media.

### 4.2. Maintaining Cell Culture

The SH-SY5Y cells (CRL-2266, American Type Culture Collection [ATCC], Manassas, VA, USA) were grown in Dulbecco’s Modified Eagle Medium (DMEM; 11-320-082, Thermo Fisher Scientific) supplemented with 10% Gibco Fetal Bovine Serum (FBS; 26140079, Thermo Fisher Scientific) and a 1% antibiotic cocktail of penicillin-streptomycin (1670249, MP Biomedical, Solon, OH, USA) as recommended by ATCC [63,64]. Cells were incubated in a 37 °C humidified incubator in a 5% CO_2_ atmosphere. Adherent cells were dissociated using 0.25% trypsin/EDTA, resuspended, counted, and plated according to use. The SH-SY5Y cells were tested at passages 5 to 12 after receipt from ATCC.

### 4.3. Dose–Response Curves

The 24 h cytotoxicity of the divalent metals was determined via viability assessment using a tetrazolium compound (3-(4,5-dimethylthiazol-2-yl)-5-(3-carboxymethoxyphenyl)-2-(4-sulfophenyl)-2H-tetrazolium, MTS; G3581, Promega, Madison, WI, USA). Cells were grown at 2.4 × 10^5^ cells/well on a 24-well plate and incubated overnight to adhere. Cells were inoculated with 7 different concentrations ranging from approximately 100% viability to 0% viability, incubated for 24 h, and then washed with 1 × phosphate buffer solution (PBS; 10010-023, Thermofisher Scientific). The absorbance assay was conducted per the manufacturer’s protocol and measured at 490 nm on a Biotek Synergy H1 spectrometer (Agilent Technologies, Santa Clara, CA, USA). Viability was normalized to untreated cells and fitted to a 4-parameter logistic curve on Sigma Plot 14.0 (Systat Software, San Jose, CA, USA). The LC_10_ and LC_50_ for each metal were determined using linear extrapolation of the data points above and below 90% viability and 50% viability, respectively [65]. For visualization only, two “blank” points were added to 100% viability and 0% viability for each metal. These values were not considered when determining the LC values.

### 4.4. Oxidative Stress

Oxidative stress was measured using the Luminescent ROS-Glo H_2_O_2_ assay (Promega, #G8820). This assay measures the production of H_2_O_2_, the reactive species with the most prolonged half-life in cell culture, before and after inoculation with a LC_10_ and LC_50_ of manganese, zinc, and copper (as determined by the dose–response curve) and 100 µM 6-ODHA used as a diseased model for neurodegeneration. In addition, experimental controls were also utilized with 1% PBS (*v*/*v*) as a negative control and 50 µM menadione as a positive control for ROS. The SH-SY5Y cells were seeded at 1.6 × 10^5^ cells/well in a white 96-well plate incubated overnight for adherence. The cells were then inoculated and incubated with metal, 100 µM 6-ODHA, or the experimental control. Cells were incubated with treatment for 24 h. The assay protocol followed the manufacturer’s instructions (i.e., luminescence detection via a Biotek Synergy H1 spectrometer).

### 4.5. Dopamine Concentration

Dopamine was assessed using an enzyme linked immunosorbent assay (ELISA) measuring the cerebral dopamine neurotrophic factor (CDNF; #EH101RB, Thermo Fisher Scientific). A higher CDNF is correlated with the protection of degeneration of dopaminergic neurons in animal models for PD [39]. The cells were seeded at 5.0 × 10^5^ on a 24-well plate and incubated overnight for adherence. After adherence, the cells were inoculated with the LC_10_ and LC_50_ of manganese, zinc, and copper (as determined by the dose–response curve) and 100 µM 6-ODHA. Cells were incubated with treatment for 24 h. In addition, experimental controls were also utilized with 1% PBS (*v*/*v*) and 50 µM menadione. Post-incubation, cells were detached with trypsin-EDTA (0.25%), washed with cold PBS, and lysed with a radioimmunoprecipitation assay (RIPA) buffer and protease inhibitor cocktail on ice according to the manufacturer’s protocol. 

The Pierce bicinchoninic acid (BCA) assay quantified the total protein (Thermo Fisher Scientific #23225). ELISA was performed according to the manufacturer’s instructions (i.e., absorbance was read at 450 nm via the Biotek Synergy H1 spectrometer). Data were plotted on a 4-parameter curve, and the concentration was extrapolated from a standard curve. 

### 4.6. DNA Damage

DNA damage was assessed by detecting the oxidation in guanine species using 8-hydroxy-2′-deoxyguanosine (8-OHdG; #EIADNAD, Thermo Fisher Scientific). The cells were seeded at 5 × 10^5^ on a 24-well plate for isolation. The cells were inoculated with a LC_10_ and LC_50_ of manganese, zinc, and copper (as determined by D/R curve) and 100 µM 6-ODHA. Cells were incubated with treatment for 24 h. In addition, experimental controls were also utilized with 1% PBS (*v*/*v*). DNA isolation (lysis and purification) was performed using the PureLink gDNA Mini Kit (Invitrogen, Waltham, MA, USA #K1820-01) according to the manufacturer’s protocols. For digestion, S1 Nuclease (#EN0321, Thermo Fisher) and Calf Intestinal Alkaline Phosphatase (CIAP, #18009019, Invitrogen) were used to conduct a double enzyme digestion protocol. 

The DNA was denatured at 100 °C for 10 min and cooled on ice. A mixture of 40 mM sodium acetate and 0.4 mM ZnCl_2_ (pH adjusted to 5.0) was added to each sample with 5 U/mL of S1 Nuclease. The samples were incubated at 37 °C for 30 m. Tris buffer (1 M, pH adjusted to 7.5) and 10 U/mL of CIAP were added to remove the phosphate caps for the DNA and incubated at 37 °C for 30 min. Samples were boiled at 100 °C to inactivate CIAP and place on ice until assaying.

A competitive ELISA detected oxidized guanine species in the digested DNA samples. Protocols were performed according to the manufacturer’s instructions (i.e., absorbance was read at 450 nM via the Biotek Synergy H1 spectrometer). Data were plotted on a 4-parameter curve, and the concentration was extrapolated from a standard curve.

### 4.7. Mitochondrial Function

Mitochondrial functionalization was assessed using the Agilent Seahorse XFe96 Pro analyzer and Cell Mito Stress Test kit (Agilent Technologies, Santa Clara, CA, USA; 103015-100). Before testing, the optimum seeding density and final drug concentrations (oligomycin, FCCP, and rotenone/antimycin A) were conducted. Cells were plated at 1.6 × 10^5^ cells/well in a Seahorse XF Pro M plate. The cells were inoculated with the LC_10_ and LC_50_ of manganese, zinc and copper (determined by D/R curve) and 100 µM 6-ODHA. Cell were then incubated for 24 h. In addition, experimental controls were also utilized with 1% PBS (*v*/*v*) and 50 µM menadione (not reported). The Mito Stress kit was applied using the manufacturer’s protocol with drug concentrations at 3 µM oligomycin, 2 µM FCCP, and 0.5 µM Rot/AA. 

### 4.8. Principal Component Analysis

The principal component analysis (PCA) parameters were chosen for explanatory value and to avoid redundancy. PCA was conducted in both directions, and the first two principal components were used to plot treatments and parameters. Data were processed and visualized in the R Studio version 4.2.2 (Boston, MA, USA) along with the packages tidyverse and ggpubr [66,67].

### 4.9. Statistical Analysis

All assays were conducted with three samples per assay and in triplicate. One-way analysis of variance (ANOVA; alpha = 0.05) was performed for each dataset for comparison with untreated cells and 100 µM 6-ODHA. Equality of variances was confirmed using the Brown–Forsythe test and Bartlett’s test. Statistical analysis was performed in Prism 9.5.0 (GraphPad, San Diego, CA, USA). Mitochondrial data were assessed using the Seahorse Report Generator. Treatments were compared using unpaired *t*-tests in the R statistical software. Asterisks or plus signs indicate *p*-value ranges: * 0.01–0.05, ** 0.001–0.01, *** 0.0001 to 0.001, **** <0.0001.

## 5. Conclusions

Our study employs a neuronal monoculture to investigate whether idiopathic metal-induced neurodegeneration operates through similar pathways as a well-established chemically induced PD model to induce neurotoxicity. Manganese, zinc, and copper are most utilized in occupational settings and likely present as lifetime or chronic exposure. Our assessment concluded that manganese and copper exhibited similar responses to 6-ODHA, a well-established model for PD. The weight of evidence does not suggest that they are replaceable models but rather induce similar pathogenesis, which leads to cytotoxicity. The current literature contains significant data associating manganese toxicity and PD. Still, it lacks data on zinc, which had a profound cytotoxic effect in our study and indicated a different mechanism from the other metals. There is insufficient evidence to properly understand the mechanism of metal-induced neurodegeneration, especially with the current “disease” models comparing toxicity to 6-ODHA. It is recommended that these metals be analyzed separately with additional gene analysis data to determine whether parkinsonism pathology is expressed. The lack of properly characterized models poses a vulnerability for the agricultural, mining, and manufacturing sectors. The current risk focuses on manganese and lead when the risk extends further to more commonly recognized metals.

## Data Availability

Data are available upon request.

## Supplementary Materials

The supporting information can be downloaded at https://www.mdpi.com/article/10.3390/ijms242216129/s1.
