# Supplementary material for: Evaluating Manganese, Zinc, and Copper Metal Toxicity on SH-SY5Y Cells in Establishing an Idiopathic Parkinson’s Disease Model
Source: Int J Mol Sci. 2023 Nov 9;24(22):16129. doi: 10.3390/ijms242216129 (PMC10671677; doi:10.3390/ijms242216129)

Supplemental Information

Supplemental Table S1. LC_{10} and LC_{50} molarity values to determine finalized dosing averages for Manganese Chloride, Zinc Chloride, and Copper Chloride after 24 h exposure on SH-SY5Y cells.

	$MnCl_2$ (µM)		$ZnCl_2$ (µM)		$CuCl_2$ (µM)	
	LC_{10}	LC_{50}	LC_{10}	LC_{50}	LC_{10}	LC_{50}
**1**	*12.32*	*142.96*	*323.16*	*338.15*	*13.75*	*758.34*
**2**	*10.82*	*137.77*	*302.48*	*318.82*	*13.33*	*692.85*
**3**	*13.55*	*148.80*	*293.86*	*313.74*	*10.43*	*716.81*
**AVG ± STDEV**	12.23 ± 1.37	143.18 ± 5.52	306.50 ± 15.06	323.60 ± 12.88	13.17 ± 2.66	722.67 ± 33.14

Supplemental Figure S1. Live metabolic oxygen consumption rate and extracellular acidification rate after 24 h treatments

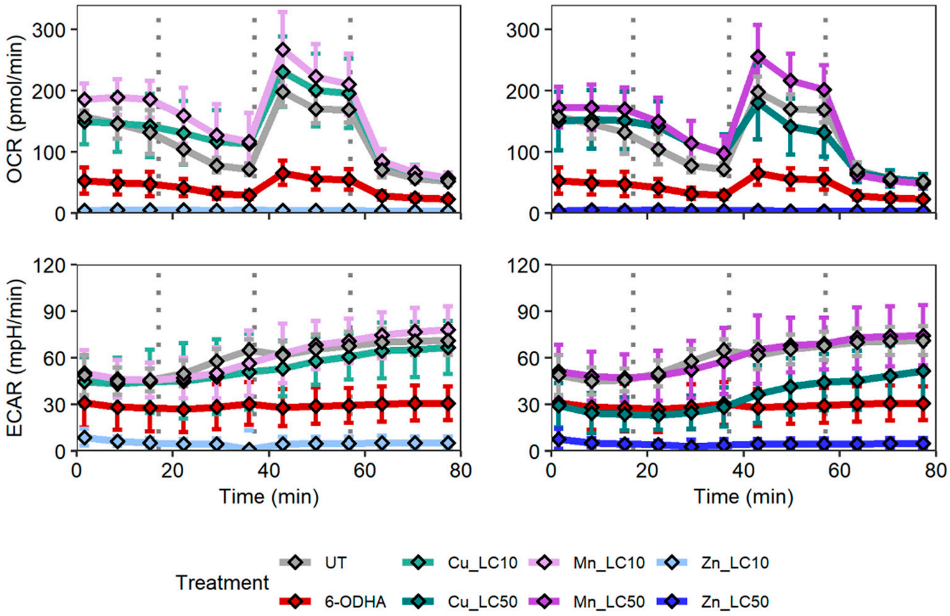

Supplement: Supplementary file 1 [file ijms-24-16129-s001.zip › ijms-2665653-supplementary.pdf]
